# Harnessing endoscopic ultrasound-guided radiofrequency ablation to reshape the pancreatic ductal adenocarcinoma microenvironment and elicit systemic immunomodulation

**DOI:** 10.37349/etat.2024.00263

**Published:** 2024-08-15

**Authors:** Vishali Moond, Bhumi Maniyar, Prateek Suresh Harne, Jennifer M. Bailey-Lundberg, Nirav C. Thosani

**Affiliations:** NGO Praeventio, Estonia; ^1^Department of Internal Medicine, Saint Peter’s University Hospital/Robert Wood Johnson Medical School, New Brunswick, NJ 08901, USA; ^2^Department of Anesthesiology, Critical Care and Pain Medicine, McGovern Medical School, University of Texas Health Science Center, Houston, TX 77030, USA; ^3^Graduate School of Biomedical Sciences, The University of Texas MD Anderson Cancer Center, University of Texas Health Science Center, Houston, TX 77030, USA; ^4^Department of Gastroenterology and Hepatology, University of Texas Health Science Center, Houston McGovern Medical School, Houston TX 77030, USA; ^5^Division of Elective Surgery and Interventional Gastroenterology, Department of Surgery, University of Texas Health Science Center, Houston TX 77030, USA

**Keywords:** Pancreatic ductal adenocarcinoma, EUS-RFA, radiofrequency ablation, tumor microenvironment, abscopal effect

## Abstract

Pancreatic ductal adenocarcinoma (PDAC) is characterized by poor prognostics and substantial therapeutic challenges, with dismal survival rates. Tumor resistance in PDAC is primarily attributed to its fibrotic, hypoxic, and immune-suppressive tumor microenvironment (TME). Endoscopic ultrasound-guided radiofrequency ablation (EUS-RFA), an Food and Drug Administration (FDA)-approved minimally invasive technique for treating pancreatic cancer, disrupts tumors with heat and induces coagulative necrosis, releasing tumor antigens that may trigger a systemic immune response—the abscopal effect. We aim to elucidate the roles of EUS-RFA-mediated thermal and mechanical stress in enhancing anti-tumor immunity in PDAC. A comprehensive literature review focused on radiofrequency immunomodulation and immunotherapy in pancreatic tumors to understand the pathophysiological mechanisms of RFA and its effect on the TME, which could prevent recurrence and resistance. We reviewed clinical, preclinical, and *in vitro* studies on RFA mechanisms in pancreatic adenocarcinoma, discussing the unique immunomodulatory effects of EUS-RFA. Recent findings suggest that combining RFA with immune adjuvants enhances responses in pancreatic adenocarcinoma. EUS-RFA offers a dual benefit against PDAC by directly reducing tumor viability and indirectly enhancing anti-tumor immunity. Observations of neutrophil-mediated immunomodulation and programmed cell death ligand 1 (PD-L1) modulation support integrating EUS-RFA with targeted immunotherapies for managing pancreatic adenocarcinoma. Integrating EUS-RFA in PDAC treatment promises direct cytoreduction and synergistic effects with molecular targeted therapies. Prospective clinical trials are crucial to assess the efficacy of this combined approach in improving outcomes and survival rates in advanced PDAC cases.

## Introduction

Pancreatic ductal adenocarcinoma (PDAC) represents a dominant and particularly aggressive form of pancreatic cancer, primarily due to diagnostic delays and suboptimal treatment efficacy. It predominantly arises in the exocrine tissue and is implicated in over 90% of pancreatic cancer diagnoses. Despite advances in understanding PDAC’s genetic characteristics, subtypes based on transcriptomic and genetic profiling, pathobiology, and therapeutic innovations, it has an average 5-year survival rate of 12%. It is anticipated to become the second leading cause of cancer–related mortality by 2030 [1, 2].

An immune suppressive environment that supports regulatory T cells (Tregs), hinders the function of cytotoxic T lymphocytes (CTLs), and facilitates tumor immune evasion is characteristic of the locally advanced pancreatic adenocarcinoma (LAPC) milieu [3]. Consequently, developing new and potent treatment strategies is crucial for improving LAPC outcomes [4]. Currently, standard PDAC treatments, including chemotherapy, radiation, and surgery, have been the mainstay for over two decades. Despite their relative effectiveness, this approach has not substantially improved overall survival (OS), mainly due to chemotherapy resistance [5].

Recent advances in endoscopic therapy could offer an additional option for treatment. Techniques such as alcohol injection, photodynamic therapy, and laser ablation, guided by percutaneous and endoscopic ultrasound (EUS), are being investigated for treating pancreatic lesions [6]. These methods are minimally invasive, increasing their practicality and potentially offering a safer alternative for patients unsuitable for surgery. EUS-guided radiofrequency ablation (EUS-RFA) represents a more recent approach that is FDA-approved for treating several gastrointestinal malignancies and is currently accessible [7]. This less invasive technique allows for outpatient care, significantly reducing morbidity compared to conventional surgery. These advancements highlight EUS’s potential as an effective, minimally invasive tumor treatment modality [8].

RFA is a minimally invasive modality leveraging high-frequency electrical currents for targeted cytoreduction of neoplastic cells, simultaneously conserving adjacent healthy tissue architecture [9]. Clinical deployment of RFA has been corroborated with an admirable safety profile, exemplified by diminished perioperative morbidity, mortality indices, and economic advantage [10–15]. The therapeutic spectrum of RFA spans a diversity of oncological pathologies, including primary and metastatic lung neoplasms, where it has demonstrated efficacy in ensuring adequate tumor clearance while maintaining pulmonary function [16]. Specifically for pancreatic adenocarcinoma, RFA is distinguished by its negligible induction of complications such as intraabdominal adhesions, positioning it as a potential adjunct to enhance survival outcomes in the preoperative setting [17].

In this review, we aim to discuss the published pathophysiological mechanism of RFA-induced alterations to the tumor microenvironment (TME), which may allow researchers to prevent PDAC recurrence and resistance. We reviewed the literature from several databases and conference proceedings including PubMed, EMBASE, and Web of Science databases (earliest inception to March 2024) for clinical, *in vivo*, and *in vitro* studies investigating the utility of implementing EUS-RFA in the management of PDAC.

## Mechanisms of RFA in PDAC: thermal injury and immune modulation?

RFA harnesses electromagnetic energy to induce controlled thermal injury within targeted tissues. The procedure of EUS-RFA involves delivering an alternating current between 350–500 kHz, which is incidentally the same frequency range used for radio broadcasts, directly to the target tissue through a specialized electrode positioned at the endoscope’s tip. The “monopolar RFA” setup comprises a closed-loop circuit with a radiofrequency generator, an insertion electrode needle, the patient’s body, and a dispersive ground pad. This electrode introduces focused energy and precipitating localized high-current density resulting in thermal elevation. The ground pad, essential to the circuit, disperses this energy across a broad area, mitigating cutaneous thermal damage [18]. Alternatively, “bipolar RFA” utilizes two juxtaposed electrodes, dispensing with the ground pad, which confines current flow and heat generation to a specified locale, enhancing the precision of tissue ablation by minimizing the perfusion-mediated cooling effect. This methodology yields a more rapid and localized thermal impact on the target area. RFA energy is well-established in clinical practice for its reliability and safety in thermal ablation procedures [19]. RFA’s mechanism of action involves high-frequency alternating currents that generate thermal energy and, in the process, cause direct thermal destruction of the tumor, resulting in localized coagulative necrosis within the tumor, releasing substantial cellular debris. The technique’s minimally invasive nature and favorable tolerance profile underscore its utility in modern therapeutic strategies for tissue ablation [20].

RFA is adaptable to lesion location and type, utilizing percutaneous image-guided delivery for hepatocellular carcinoma and liver metastases, intraoperative application for internal lesions, endobiliary routing for inoperable biliary or pancreatic cancers with obstruction, and endosonographic targeting for pancreatic or adjacent anomalies. Diverse EUS-RFA probes for pancreatic lesions have been documented: a 19 G EUS-Fine needle biopsy (FNA) needle electrode, the Habib™ EUS-RFA catheter, a bipolar hybrid cryotherm probe, and the EUS-RFA electrode, each with unique features like internal cooling systems in the latter two to prevent electrode charring for optimal heat conduction [7, 21, 22]. These devices are categorized into “through-the-needle” and “EUS-FNA needle-type”, with the needle-type probes being rigid and insulated except at the terminal tip, varying in length. These probes, connected to advanced generators, facilitate precise energy delivery to lesions. Table 1 lists current clinical trials incorporating EUS-RFA into the clinical management of pancreatic cancer.

**Table 1:** List of current clinical trials incorporating EUS-RFA into the clinical management of pancreatic adenocarcinoma

**Study title for RCT**	**NCT number**	**Conditions**	**Interventions**	**Location**
EUS-RFA for pancreatic neoplasms	NCT03218345	Pancreatic neoplasms	Procedure: EUS-RFA	Hong Kong, China
EUS-RFA for unresectable pancreatic cancer	NCT04310111	PDAC	Procedure: EUS-RFA	China
EUS-RFA for unresectable PDAC	NCT03772756	Pancreatic adenocarcinoma non-resectable	Procedure: EUS guided RFA Radiation: chemoradiotherapy	China
EUS-RFA for treatment of PDAC	NCT05723107	Pancreatic cancer	Drug: chemotherapy Device: EUS-RFA	NY, USA
Evaluation of safety and feasibility of EUS-RFA for solid pancreatic neoplasms	NCT03435770	Pancreatic neoplasms	Device: EUS-RFA needle	Singapore
Evaluation of EUS-RFA for the management of pancreatic tumors, ERASE Study	NCT05961982	Pancreatic neoplasm	Procedure: EUS-guided fine-needle aspiration Procedure: EUS-RFA	Ohio, USA
A single-arm phase II study to evaluate the safety and efficacy of combination systematic chemotherapy and multiple rounds of EUS-RFA in pancreatic cancer	NCT04990609	PDAC	Device: EUS-RFA Drug: neoadjuvant chemotherapy (NAC)	Houston, USA
EUS-RFA of not-resectable pancreatic cancer	NCT04164992	PDAC	Device: EUSRA electrode needle connected to a radiofrequency generator (VIVA RF Generator; STARmed, Seoul, S. Korea)	Italy
Safety and efficacy of an ablation catheter for the treatment of pancreatic premalignant cyctic lesions	NCT03417843	Pancreatic cancer Pancreatic neuroendocrine tumor Pancreatic Cyst	Device: EUSRA RF electrode	Israel
Trial comparing EUS-RFA vs. EUS-guided celiac plexus neurolysis	NCT03152487	Pancreatic adenocarcinoma Pancreatic neoplasms	Celiac plexus neurolysis RFA	Orlando, USA
EUS-coeliac plexus block vs. RFA in pain relief of patients with malignancy	NCT04809935	Cancer of pancreas Pancreatic neoplasms	Drug: 98% dehydrated alcohol Device: 19 G EUSRA needle, Taewoong Medical, Korea	Hong Kong, China
Efficacy and safety of RFA in pancreatic neuroendocrine and cystic tumor	NCT02330497	Pancreatic tumor Endocrine tumor Neoplasms, cystic, mucinous, and serous	Procedure: RFA under EUS	France
EUS RFA, database repository	NCT04693754	Pancreatic neoplasm	Procedure: RFA under EUS	Indiana, USA

RCT: randomized clinical trial; EUS-RFA: endoscopic ultrasound-guided radiofrequency ablation; PDAC: pancreatic ductal adenocarcinoma; FNA: fine needle biopsy

Goldberg et al. [8] documented the first use of EUS-RFA in porcine models, confirming imaging-pathology consistency for lesions over 5 mm, with computed tomography (CT) post-RFA revealing interstitial hemorrhage. Minor complications included gastric and intestinal burns due to electrode misplacement and a single case of elevated lipase with resultant pancreatitis [8]. In animal models of pancreatic RFA, short-term results appeared safe as most rodent or porcine patients survived unharmed until euthanized per the research protocol. However, its clinical translation faces obstacles due to endoscopic and technological complexities. Given that many surgeons lack training in pancreatic ablation and that conventional surgical or laparoscopic methods have notable limitations and higher postoperative complication rates, endoscopic RFA emerges as a preferable option. Thus, endoscopic RFA may be the ideal approach compared to traditional open or laparoscopic pancreatic ablation, in which postoperative complication rates approach 25% [23]. It also benefits from simultaneous EUS imaging, offering improved visualization. The EUS method has yielded positive outcomes with low complication rates in treating pancreatic neuroendocrine tumors with less than (< 10% complication rate) [24]. According to recent findings by Barthet et al. [25], 65% of patients undergoing EUS-RFA for neuroendocrine cystic neoplasms experienced a complete resolution of the targeted lesion. These findings advocate EUS-RFA as a viable and lasting treatment for pancreatic neuroendocrine tumors (< 3 cm) and select cystic neoplasms.

## The role of ablation in immunomodulation: local vs. systemic

Recently, RFA has been recognized for its role in the palliative care of malignant biliary obstructions [26, 27]. Evidence suggests that RFA offers symptom relief and may extend survival [28, 29]. While multi-agent chemotherapy is the current gold standard for preoperative treatment, a significant number of PDAC patients are ineligible for surgery that could potentially be curative. Both chemotherapy and chemoradiation have been utilized for borderline resectable and locally advanced PDAC with variable outcomes [30]. However, current treatments, including chemotherapy, radiation, and immune checkpoint blockade (ICB), have proved to be largely ineffective against PDAC [31, 32].

**Figure 1:**
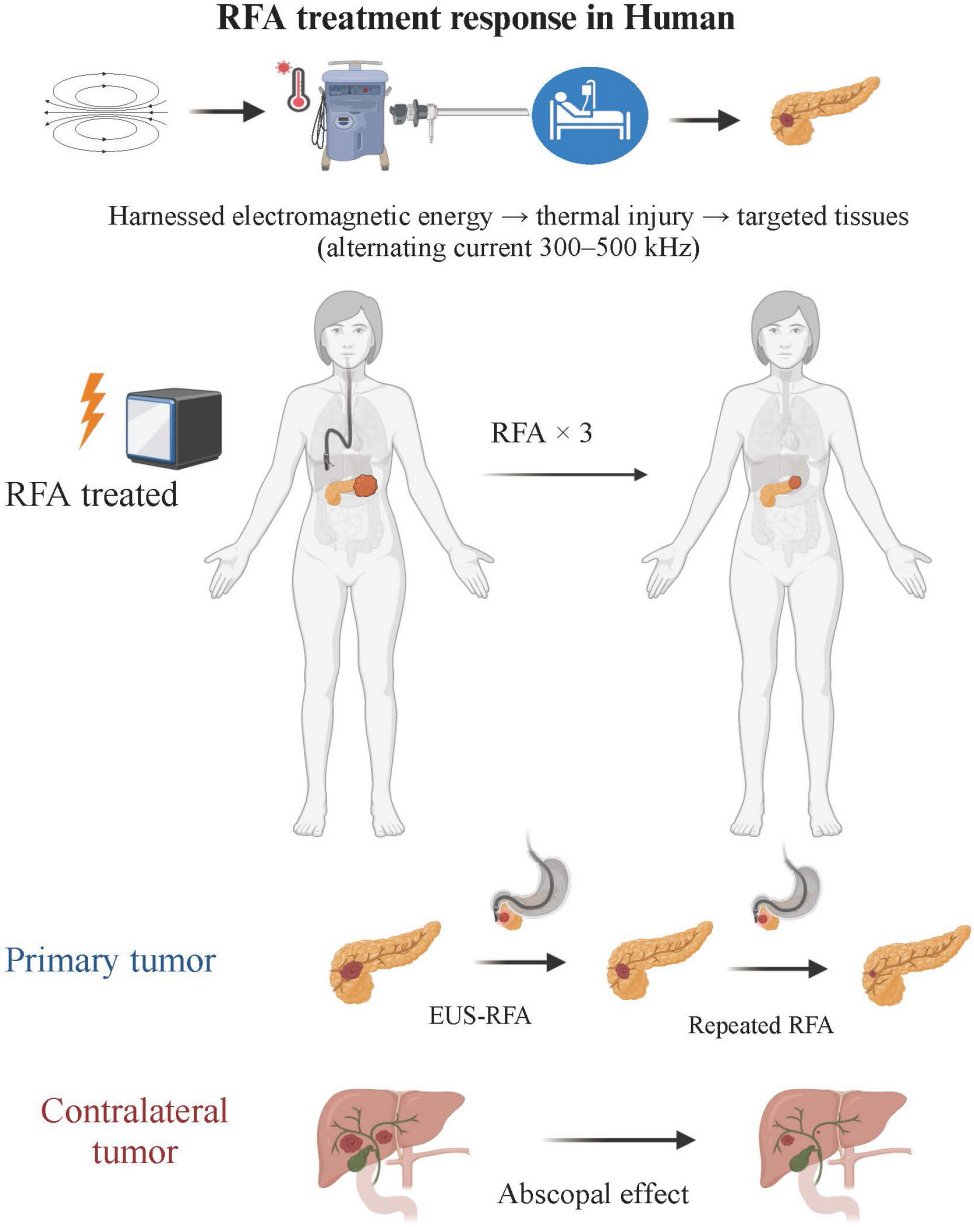
EUS-RFA technique and treatment response in human. This figure illustrates the application of the EUS-RFA technique for the treatment of tumors. The EUS-RFA procedure involves delivering an alternating current to the target tissue, resulting in thermal elevation and localized coagulative necrosis within the tumor. The figure also discusses the abscopal effect observed in humans, where localized RFA treatment leads to a systemic immune response and regression of untreated tumors. Overall, this figure provides a comprehensive overview of the RFA technique and its treatment response in human patients (Created with BioRender.com). EUS-RFA: endoscopic ultrasound-guided radiofrequency ablation

**Figure 2:**
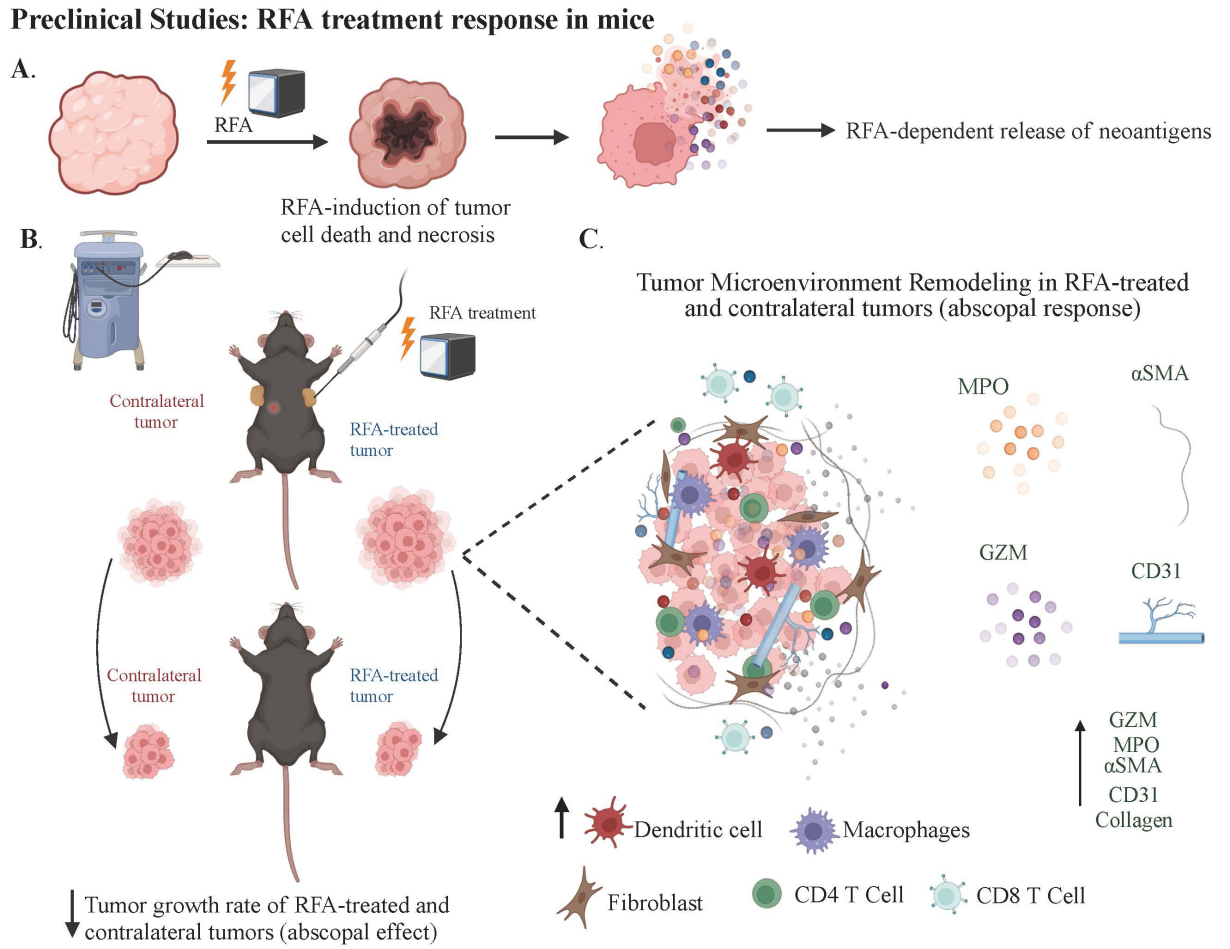
RFA treatment response in mouse models and resulting TME modulation. A) The RFA procedure leads to localized coagulative necrosis within the tumor and release of tumor antigens; B) it highlights the observed abscopal effect of the RFA therapy in mouse models. This figure presents the immune response elicited by RFA in the primary TME and its potential impact on distant metastatic tumors. RFA induces substantial modifications in the TME, influencing local and systemic immune landscapes; C) the application of EUS-RFA results in extensive necrosis and reduced viability of epithelial cells in treated tumors. Additionally, the ablation site exhibits significant infiltration of inflammatory cells (MPO+), vascular cells (CD31+), and cytotoxic T-cells (Granzyme B+), both within and surrounding the ablation area (Created with BioRender.com). TME: tumor microenvironment

With the advent of new medical technologies, local ablative treatments like EUS-RFA are gaining traction in PDAC management for several potential benefits: direct tumor ablation via coagulative necrosis, increased effectiveness of chemotherapy as tumors may allow better penetration of systemic therapies, and potential amplification of systemic anti-tumor immunity through recognition of necrotic tumor debris by the immune system (Figure 1). A summary of published clinical data incorporating EUS-RFA for the management of PDAC is shown in Table 2 [7, 14, 26, 27, 33–39]. Studies in both animal models and cancer patients have demonstrated that RFA not only locally disrupts tumors through heat but also induces coagulative necrosis. This necrosis releases cellular debris, providing tumor antigens that can activate an adaptive immune response to attack both the local and distant tumor sites, an outcome known as the abscopal effect (Figure 2) [40, 41]. Thosani et al. [38] proposed the systemic immune activation observed following tumor ablation, explaining prolonged survival in a patient with stage IV ampullary carcinoma surviving 73 months post-diagnosis. While the underlying tumor biology is crucial, the possibility remains that RFA acted to prime the immune system for a systemic therapeutic effect.

**Table 2:** A summary of published clinical data incorporating EUS-RFA for the management of pancreatic adenocarcinoma

**Study**	**Study details**	**Patients, (*n*)**	**RFA session, (*n*)**	**Male/Females, (*n*)**	**Mean age (years)**	**Tumor location (*n*)**	**Cancer stage (*n*)**	**Mean size (range in mm)**	**Any decrease in tumor size, [*n* (%)]**	**Catheter/Needle used**	**RFA energy**	**Use of adjunct chemotherapy, (*n*)**	**If yes, which chemotherapy (*n*)**	**Technical success [*n* (%)]**	**Clinical success, [*n* (%)]**	**Adverse events per session/patient, [*n* (%)]**	**Mean follow-up in months**	**Survival after RFA in months**
Arcidiacono et al. [34], 2012	Prospective, September 2009–May 2011, multiple centre, Germany and Italy	22	22	11/11	61.9	Head (16), uncinate (2), body (4)	Locally advanced (22)	35.7 (23–54)	6 (37.5)	22/25-gauge needle	18 W	yes	Gemcitabine-based chemotherapy, chemoradiation therapy (6)	16/22 (72.7)	NR	8/22 (36.4)	3	5.6 (1–12)
Bang et al. [26], 2019	Prospective, single-blind, randomized trial, Florida, USA	12	12	7/5	62.8 ± 13.7	Head and uncinate (8), body and tail (4)	Locally advanced (5), metastasis (7)	29.6 (22.5–35.0)	NR	19-gauge FNA needle	10 W	yes, 6 patients	NR	12/12 (100)	NR	5/12 (41.6)	1	NR
Crinò et al. [35], 2018	Retrospective, single-centre, November 2016 and August 2017, Italy	8	8	5/3	67	Pancreas head (3), body (3), and uncinate process (2)	Locally advanced (7)	36 (22–67)	8/8 (100)	18-gauge needle	30 W	yes, 6 patients	Folfirinox + Radiotherapy, Folfirinox only, Gemcitabine	8/8 (100)	8/8 (100)	3/7 (42.8)	6.1	6
Kongkam et al. [14], 2023	Prospective, single-centre, July 2017– August 2018, Thailand	10	30	4/10	66.3 ± 10.8	Head (5), body (11), neck (12), and uncinate process (2)	Stage III 1 (7); stage III b 6 (21); stage IV 10 (72)	62.2 ± 21.0	10/10 (100)	19-gauge needle	50 W	yes, 10 patients	Gemcitabine alone (6), Nab-paclitaxel plus gemcitabine (3), and mFOLFIRINOX (1)	10/10 (100)	10/10 (100)	1/14 (7)	6	NR
Oh et al. [36], 2022	Prospective, single-centre, May 2016– June 2019, South Korea	22	107	13/9	60.5 (56.25–68.75)	Head (14), body (4), tail (3), metastasis (1)	Locally advanced (14), metastatic (8)	38 (32.75–45)	NR	19-gauge needle	50 W	yes, 22 patients	Gemcitabine‐based chemotherapy	22/22 (100%)	2/22 (9)	4/107 (3.7)	21.23 (10.73–27.1)	24
Paiella et al. [37], 2018	Retrospective, single-centre, October 2008 –January 2015, Germany	30	30	20/10	64 (44–81)	Head (23), body and tail (7)	Locally advanced (30)	35 (20–60)	NR	NR	NR	yes, 17 patients	Chemotherapy (17) FOLFIRINOX (6) Gemcitabine/oxaliplatinum (4) Nab-paclitaxel/gemcitabine (2) Not known (5)	30/30 (100%)	NR	4/30 (13.3)	15 (4–38)	15
Scopelliti et al. [27], 2018	Prospective, single centre, February 2016– October 2016, Italy	10	10	7/3	62(50–74)	Head (4), body (6)	Locally advanced (10)	49.2 (25–75)	10 (100)	18-gauge needle	30 W for lesions > 3 cm; 20 W for < 3 cm	yes	FOLFIRINOX (4), gemcitabine/Nab-paclitaxel (2), Gemcitabine (2), GemOx (2)	10/10 (100%)	NR	4/10 (40)	1	NR
Song et al. [7], 2016	Prospective, single-centre, February 2013–March 2014, South Korea	6	8	1/5	62 (43–73)	Head (4), body (2)	Locally advanced (4), metastasis (2)	48 (30–90)	NR	18-gauge needle	20–50 W	yes, 3 patients	Gemcitabine (3)	6/6 (100%)	NR	2/6 (25)	4.2	NR
Thosani et al. [38], 2022	Prospective, single-centre, October 2016 –March 2018, Texas	10	22	7/3	62	Head (4), neck (2), body (2), and tail (2)	Locally advanced (7), metastasis (3)		7/10 (70)	19/22-gauge	10–15 W	yes	mFOLFIRINOX (20), gemcitabine/abraxane (1), both mFOLFIRINOX and gemcitabine/abraxane (6), mFOLFIRINOX and gemcitabine/Abraxane + cisplatin (1)	10/10 (100%)	10/10 (100)	0	81	20.5 (9.93–42.2)
Wang et al. [39], 2021	Retrospective, single centre, November 2013– November 2018, China	11	26	6/5	64.7 (42–83)	Head (4), neck (3), body (3), tail (1)	Locally advanced (7), metastasis (4)	28 (17.2–38)	2 (18.2)	22-gauge	5–10 W	yes, 1 patient	NR	11/11 (100%)	NR	2/11 (18.1)	5.2	5.2

*n*: number; mm: millimetre; W: watts; EUS-RFA: endoscopic ultrasound-guided radiofrequency ablation

Other studies have shown that combining ICB therapy, explicitly targeting PD-L1 upregulated by RFA, with RFA itself, can significantly halt tumor progression in treated and distant tumors [42]. Moreover, studies also suggest radiation therapy can alter the abscopal mechanism, driving tumor-infiltrating neutrophils toward an anti-tumor phenotype [43]. Future preclinical trials are aimed at assessing the efficacy of RFA with adjunctive ICB treatments to determine their viability for clinical application, particularly in restraining tumor growth and enhancing survival in PDAC patients, with a focus on those with advanced or metastatic disease where new treatments are crucial to improve prognoses.

## RFA reduces PDAC progression *in vivo* and increases pro-inflammatory mediators

Tumor growth dynamics in mice subjected to RFA have been noted to have suppression of tumor expansion in RFA-treated sites as opposed to sham-treated controls, with a pronounced abscopal effect evident in a majority of the non-RFA-treated tumors (Figure 2). These findings indicate a systemic anti-tumor response is elicited by localized RFA treatment without significant weight changes in the subjects, indicating a lack of systemic toxicity [42, 44]. At the same time, histological analysis showed that RFA-treated tumors exhibited an increased necrotic area. Additionally, staining for apoptotic and cytotoxic markers, such as cleaved caspase 3 and Granzyme B (GZM), revealed significantly higher staining in RFA-treated tumors, suggesting an enhanced anti-tumor response. Antibody array analysis of tumor tissues revealed upregulation of immune modulators such as complement function (C5/C5a), interleukin (IL)-23, and chemokine (C-X-C motif) ligand 12 (CXCL12) post-RFA, with a corresponding increase in chemotactic chemokines in the serum, further implicating the immune system’s local and systemic role in this process. CXCL13 levels were significantly elevated, corroborating its importance in immune response facilitation and the potential mechanistic involvement in the abscopal effect [42].

RFA has also been shown to increase neutrophil infiltration, causing an alteration in the tumor immune microenvironment. This study identified the neutrophils as pro-inflammatory, as evidenced by co-localization with myeloperoxidase (MPO). Restructuring of the TME by RFA was also highlighted by enhanced expression of α-smooth muscle actin (αSMA), collagen, and cluster of differentiation 31 (CD31) within both treated and distant non-treated tumors, suggesting a global effect of RFA on the TME [42]. Further investigations into the role of neutrophils in the abscopal effect were studied using Imaging mass cytometry (IMC), which revealed a substantial presence of lymphocyte antigen 6 family member G (Ly6G) CD11b CD44 neutrophils in the vicinity of the necrotic core. These neutrophils were found to closely associated with αSMA+ myofibroblasts, pancytokeratin (PanCK+) tumor cells, and various immune cell subsets, indicating a concerted interplay within the TME. A neutrophil depletion study provided further insight into their critical role in mediating the abscopal effect. Depletion of neutrophils led to increased tumor growth in distant non-RFA-treated tumors and affected fibrotic responses, suggesting a direct or indirect regulatory function of neutrophils on fibroblasts and the adaptive systemic abscopal effect [45].

In sustained tumor control, integrating *in vivo* ICB therapy with RFA was evaluated for its impact on long-term tumor progression. Initial findings showed continued growth suppression in both RFA and non-RFA tumors, with PD-L1 expression elevated in non-RFA tumors. These preliminary observations strengthen the premise for future preclinical and clinical trials employing RFA in combination with ICB. These studies should also include an in-depth analysis of these therapeutic combinations beyond safety and survival to understand better the mechanistic underpinnings of these emerging therapeutic considerations in the clinical management of PDAC [44, 46].

## RFA elevates adenosine and inosine production in KPC subcutaneous tumor

Extracellular adenosine (eADO) metabolic conversion from adenosine monophosphate (AMP) and signaling through adenosine (ADO) receptors has been investigated due to the immune suppressive roles of elevated eADO signaling in tumor progression [47–49]. Recent studies that performed high-performance liquid chromatography (HPLC) analyses indicated an acute elevation of AMP in tumor tissues at four days post-RFA, which normalized by day 10. Conversely, serum AMP levels were upregulated at ten days in both sham and RFA-treated mice without discernible differences between groups. Tumor ADO levels remained significantly elevated in RFA-treated mice 4 days and 10 days post-treatment. In contrast, a significant rise in serum eADO was observed only at day four post-RFA, indicating a differential temporal response in local vs. systemic ADO levels [44].

Inosine (INO) levels were augmented in tumor tissues four days post-treatment in sham and RFA groups, with no differences. Interestingly, serum INO decreased in the RFA group at four days post-treatment compared to sham, which inverted by day 10 with elevated levels in the RFA group during the more chronic phase [44].

These findings suggest a complex interplay between the acute and chronic phases of RFA treatment, with significant alterations in the eADO pathway components. The persistent elevation of ADO in tumor tissues post-RFA indicates its potential involvement in the local tumor response to ablation therapy. In contrast, the temporal dynamics of serum INO suggest a role in mediating systemic responses, potentially contributing to tumor growth or immune suppression at later stages post-RFA. The study underscores the importance of considering local and systemic biochemical changes when evaluating the efficacy and potential resistance mechanisms to RFA in PDAC treatment.

## RFA increases neutrophil infiltration and induces systemic TME remodeling

Recent research has elucidated the significant role of neutrophils in mediating anti-tumor immunity, particularly in the context of RFA [44]. Neutrophils, specifically tumor-associated neutrophils (TANs), can exhibit a dual nature, either promoting or inhibiting tumor growth based on their activation state. The role of neutrophils in enhancing anti-tumor immunity has been demonstrated through their production of reactive oxygen species (ROS) and cytokines, as well as their ability to recruit and activate other immune cells [44].

Faraoni et al. [44] highlighted that neutrophils play a pivotal role in mediating the abscopal effect observed after RFA treatment in PDAC. Their study demonstrated that RFA-treated tumors exhibited increased neutrophil infiltration, which was associated with a systemic anti-tumor response. This was evidenced by the presence of pro-inflammatory markers and the activation of CTLs and natural killer (NK) cells in both treated and untreated tumor sites [44]. Neutrophils release various chemokines and cytokines that attract CTLs and NK cells to the tumor site. Takeshima et al. [43] showed that radiation-induced anti-tumor immune responses were potentiated by granulocyte-colony stimulating factor (G-CSF), which enhances neutrophil activity. This potentiation was linked to the recruitment of CTLs and the modulation of the TME to favor immune cell infiltration.

Multiple intersecting pathways are involved in neutrophil-mediated anti-tumor immunity. Neutrophil extracellular traps (NETs) and kill tumor cells and facilitate tumor antigen presentation to T cells. Neutrophils also modulate myeloid-derived suppressor cells (MDSCs) activity, which suppresses T cell function and promotes tumor growth. By inhibiting MDSCs, neutrophils enhance the overall anti-tumor response [50]. Neutrophils activate the complement system, leading to the opsonization and destruction of tumor cells and the recruitment of other immune cells [51]. Neutrophils release proteins such as MPO and elastase, which directly kill tumor cells and modulate the TME to favor immune cell infiltration [52].

Studies have shown a significant increase in IL-23 levels in PDAC tumor homogenates, indicative of active neutrophil engagement [53, 54]. Immunohistochemical (IHC) staining confirmed elevated neutrophil markers NIMPR14 and MPO, particularly in necrotic regions of both RFA-treated and untreated tumors [42]. The observed co-localization of MPO with NIMPR14 suggests these neutrophils are pro-inflammatory. Further, RFA-treated and adjacent non-treated tumors exhibited heightened αSMA, a marker of myofibroblast activity, and CD31, indicative of angiogenesis, compared to controls [42]. Moreover, Faraoni et al. [42] showed that the absence of neutrophils led to increased tumor growth in distant non-RFA-treated tumors, emphasizing the critical role of neutrophils in mediating the abscopal effect and systemic anti-tumor immunity.

These findings imply that RFA induces significant changes in the TME, influencing both local and systemic immunological landscapes, essential for tissue repair and the efficacy of subsequent immunotherapies.

## Role of neutrophils in mediating the anti-tumor effects observed in the abscopal response

Using IMC to analyze regions near the necrotic core in non-RFA-treated tumors revealed a proliferation of Ly6G CD11b CD44-positive neutrophils [42]. These neutrophils were found near αSMA-expressing myofibroblasts, PanCK-positive tumor cells, CD44-expressing tumor stem cells, and dendritic and macrophage populations, suggesting a complex, immune-rich TME. An *in vivo* neutrophil depletion study coupled with RFA ablation showed that non-RFA tumors from neutrophil-depleted mice significantly increased in size, underlining the crucial role of neutrophils in mediating the abscopal effect [42]. Furthermore, in the absence of neutrophils, an upregulation of αSMA was explicitly noted in distant non-treated tumors, hinting at neutrophils’ involvement in abscopal fibrosis, potentially through modulation of myofibroblast activity. However, CD31 expression, a marker of angiogenesis, remained unchanged across all groups. Significantly, CXCL13 levels were diminished in both serum and RFA-treated, neutrophil-depleted tumors, suggesting that a decrease in this chemokine might be linked to mitigated tumor growth and emphasizing the significance of neutrophils in the systemic anti-tumor response following RFA [42].

Another study analyzed single-cell neutrophil transcriptomes from 17 different cancer types, uncovering significant complexity with 10 distinct functional states, including roles in inflammation, angiogenesis, and antigen presentation. Notably, neutrophils in the antigen-presenting state were linked to better survival outcomes in most cancers and could be induced through leucine metabolism and histone H3K27ac modification [45]. These neutrophils were capable of initiating both (neo)antigen-specific and antigen-independent T cell responses. Furthermore, administering neutrophils or a leucine-rich diet improved the effectiveness of anti-PD-1 therapy in various murine cancer models [45]. The findings underscore the diversity of neutrophil functions across cancers and suggest therapeutic potential in using antigen-presenting neutrophils.

## Combining ICB with RFA effectively maintains tumor growth inhibition *in vivo*

**Figure 3:**
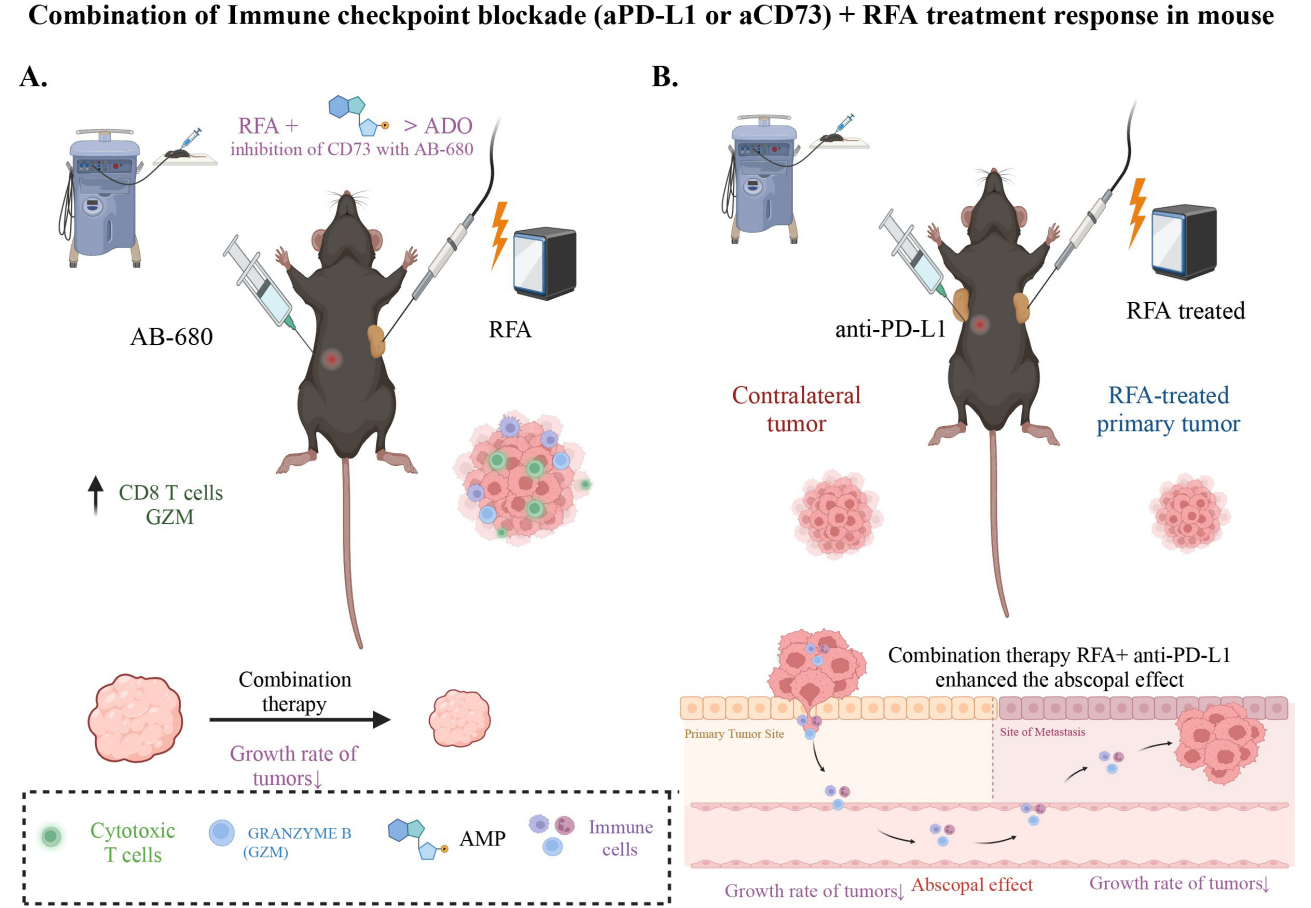
The synergistic effect of immune checkpoint blockade and RFA combination therapy. A) RFA+ inhibition of CD73 significantly reduces the growth rate of combination treated tumors compared to RFA alone. IHC analysis of RFA+ CD73 inhibitor treated tumors showed a significant increase in GZM and CD8+ T cells; B) depiction of the combination therapy of immune checkpoint blockade using anti-PD-L1 and RFA, showcasing its positive response in mouse models. RFA + aPD-L1 therapy restrains tumor growth. The abscopal effect observed in contralateral tumors suggests the potential of this treatment approach for locally advanced or distant tumors (Created with BioRender.com)

Faraoni et al. [42] revealed that long-term monitoring causes inhibition of initial tumor growth by RFA post-treatment. At the same time, IMC showed increased dendritic cells and reduced MDSCs in RFA-treated tumors, indicating an activated immune profile. Enhanced CD4 and CD8α T-cell levels in non-RFA tumors suggested a systemic anti-tumor response. Notably, the IMC data showed elevated PD-L1 expression in RFA-treated tumors, which indicates the presence of tumor-intrinsic immune resistance. Spatial analysis linked PD-L1 to tumor and stromal cells, which revealed targets for therapy [42]. Anti-PD-L1 antibody treatment post-RFA sustained the inhibition of tumor growth through day 10, demonstrating the therapeutic potential of combining RFA with ICB to enhance anti-tumor immunity and prevent tumor relapse (Figure 3). Future studies in murine models employing chemotherapy with RFA may show chemotherapy reduces post RFA PD-L1 expression.

## RFA TME and immune modulation in human pancreatic tumors

In a small clinical study of three patients with stage I and III PDAC, EUS-RFA resulted in considerable necrosis and reduced epithelial cell viability in treated tumors, mirroring preclinical results [41]. Resected tumors from a stage III patient exhibited significant infiltration of inflammatory cells (MPO+), vascular cells (CD31+), and cytotoxic T-cells (GZM+), both in and around the ablation site. Serum proteome profiling pre- and post-EUS-RFA revealed increased levels of inflammatory and immune markers (CCL5, CD40, C5/C5a, ICAM, MIF, and SERPIN). These findings corroborate the preclinical data, indicating that EUS-RFA induces local and systemic immune responses and could be a promising treatment modality for PDAC, deserving further investigation.

## Inhibition of CD73 *in vivo* prevented tumor enlargement, necrosis, and anti-tumor immunity

Studies have also shown that the ADO pathway, particularly the enzyme CD73, plays a significant role in the immunosuppressive TME [55, 56]. Blocking CD73 can enhance anti-tumor immunity by reducing ADO production and increasing the presence of cytotoxic T cells. Hay et al. [48] (2016) demonstrated that targeting CD73 in the TME with MEDI9447 improved immunotherapeutic outcomes, suggesting a potential combinatory approach with RFA to enhance its efficacy.

The upregulation of the ADO pathway following RFA treatment prompted an investigation into its potential role in the tumor regrowth noted post-treatment. Considering the ADO pathway’s implication in tumor progression and immunosuppression, it has been shown that the pathway’s activation might contribute to the resurgence of tumor growth [44]. Serum aspartate aminotransferase (AST) levels indicated lower toxicity in all RFA-treated mice, including those treated with AB-680 (a reversible and selective inhibitor of CD73), than sham-treated controls. Notably, the group treated with AB-680 exhibited no significant tumor volume increase post-RFA, suggesting a blockade of tumor re-expansion (Figure 3). In contrast, the RFA and RFA + VEH groups demonstrated considerable tumor growth post-initial acute response [44].

This outcome suggests that CD73 enzymatic activity is pivotal in the ADO pathway’s contribution to tumor relapse post-RFA treatment. The successful prevention of tumor regrowth with CD73 inhibition positions AB-680 as a potential therapeutic to enhance the efficacy of RFA, thereby mitigating the immunosuppressive microenvironment and possibly impeding tumor progression. These results substantiate the need for further research to elucidate the mechanisms by which the ADO pathway influences tumor recurrence and to validate the clinical applicability of combining CD73 inhibitors with RFA in treating PDAC.

## CD73 inhibition *in vivo* in combination with RFA impairs the ADO pathway and associated immune suppression

The role of RFA in eliciting an anti-tumor immune response in PDAC has been previously documented [42]. Analysis of human PDAC tissues has shown that elevated CD73 levels in PDAC patients correlate with poor prognosis due to increased ADO generation and decreased intertumoral CD8+ T cells [57–59]. It has been noted that treated tumors present a negative correlation in the spatial distribution of CD73 and GZM+ cells, suggesting therapies targeting CD73 may increase anti-tumor immunity throughout the cancer [42]. This indicates that some patients may have improved responses when ADO pathway activation with EUS-RFA is impaired. In a preclinical model using a cell line with high expression of CD73, CD73 inhibition during combination therapy with RFA impaired tumor enlargement and sustained RFA-induced anti-tumor immunity [44]. In the same study, the impact of RFA combined with AB-680, a CD73 inhibitor, on anti-tumor immunity was evaluated by analyzing GZM expression. IHC analysis determined the presence of GZM and CD8α positive cells post-treatment, compared to a control group of untreated standard KPC subcutaneous tumors (Figure 3).

The analysis demonstrated that RFA, in conjunction with AB-680, significantly increases the presence of GZM+ cells, which indicate cytotoxic T-cell activity, compared to both the control tumors and those treated with RFA alone. The enhanced abundance of CD8α-positive cells corroborates their likely role as the source of GZM. These results align with the observed reduction in tumor growth, suggesting a nexus between increased cytotoxicity and anti-tumor immunity and the diminished proliferation of tumor cells. Exploring the spatial relationship between CD73 expression and GZM+ cells has also revealed an inverse correlation, where areas of reduced CD73 corresponded with increased GZM+ presence, highlighting the immune suppressive role of CD73 in PDAC.

Further metabolic analysis via HPLC has shown that tumors treated with the RFA and AB-680 combination had significantly lower levels of AMP, ADO, and INO compared to RFA treatment alone [44]. This indicates the effectiveness of CD73 inhibition in conjunction with RFA in reducing tumor metabolite levels associated with immune suppression.

Through transcriptomic investigation of the enzymes involved in these metabolic pathways, it was noted that there were no significant differences in the protein and messenger ribonucleic acid (mRNA) expression levels of CD39 and Nt5E (CD73), nor in adenosine deaminase (ADA) protein expression across the treatment groups [44]. However, ADA mRNA levels were lower in the RFA and AB-680 combination group, suggesting that AB-680 may attenuate the tumor’s ability to produce immunosuppressive metabolites, especially INO, in the presence of inhibited CD73 activity [44]. This indicates that combining *in vivo* CD73 inhibition with RFA may thwart the ADO pathway’s emergence as a resistance mechanism. By reducing the tumor’s capacity to produce immunosuppressive metabolites like ADO and INO, this combination therapy may lead to heightened anti-tumor immunity, thus presenting a compelling strategy for enhancing PDAC treatment efficacy.

## Targeting the TME in combination with other target therapies

Targeted therapies in pancreatic cancer remain challenging due to the intricate signaling network and compensatory pathways of the disease, often leading to therapeutic resistance [60]. Currently, the combination of erlotinib, a tyrosine kinase inhibitor, with gemcitabine stands as the only FDA-approved targeted therapy for metastatic pancreatic cancer, providing minimal benefit [61]. The TME, particularly the cancer stroma, has been relatively understudied but is increasingly recognized as a significant factor influencing the efficacy of targeted treatments. Lonardo et al. [62] highlighted the potential of targeting the Nodal/Activin pathway to impede the self-renewal and tumorigenicity of pancreatic cancer stem cells and overcome gemcitabine resistance. However, stromal components may attenuate gemcitabine efficacy, a setback that could be countered by adding a sonic hedgehog pathway inhibitor. Further, combining mammalian target of rapamycin (mTOR) inhibitors with sonic hedgehog inhibitors has been shown preclinically to enhance disease control and increase the susceptibility of cancer stem cells to chemotherapeutic agents [63]. These insights point to a multifaceted approach that targets both cancer cells and their stromal interactions as a promising direction for advancing the treatment of pancreatic cancer.

## Conclusion

**Figure 4:**
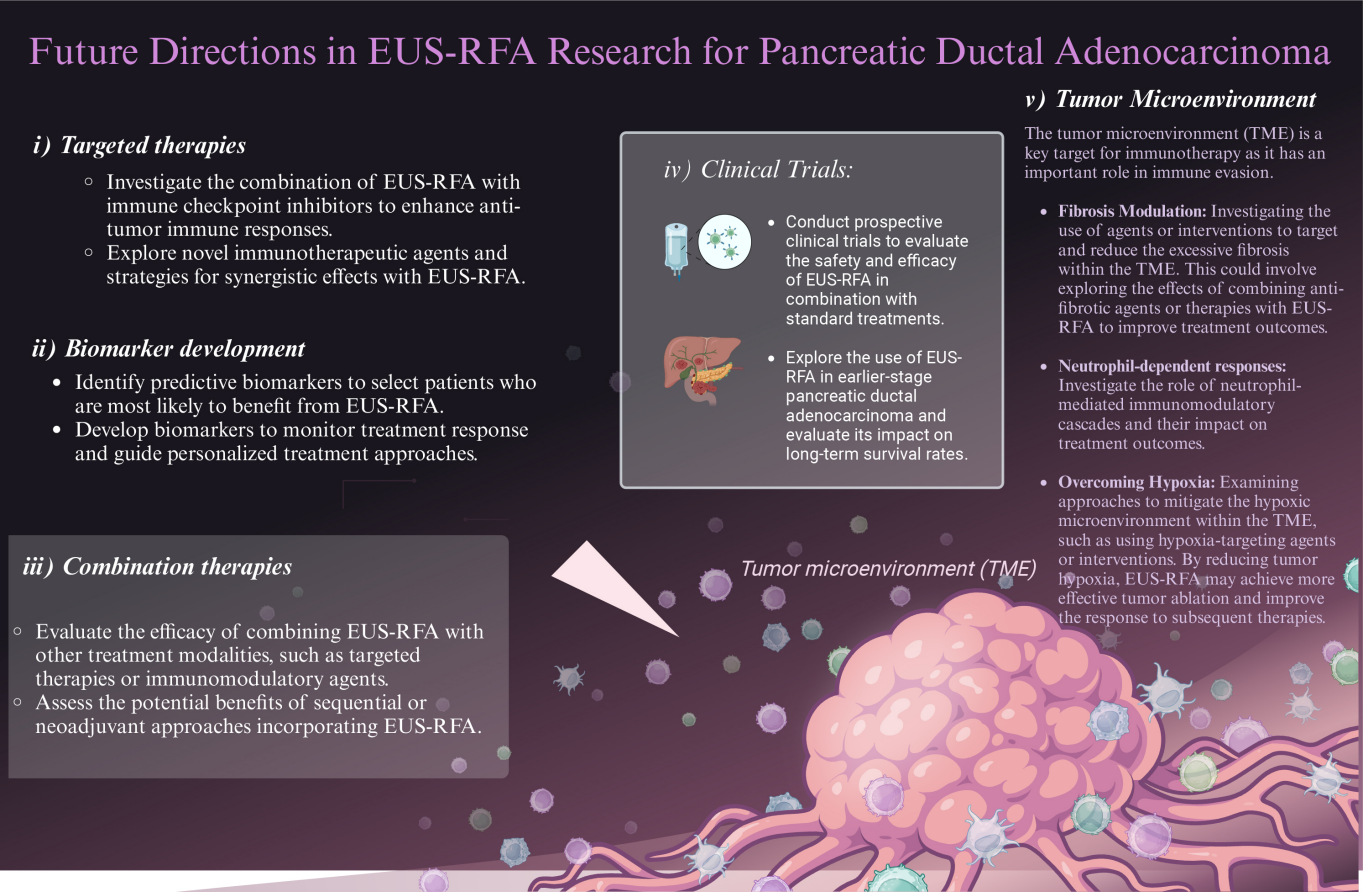
Future directions for RFA research and enhancing response and treatment in pancreatic ductal adenocarcinoma (PDAC). Visual representation of potential advancements in RFA research for the treatment of PDAC. The image showcases various strategies and techniques that could be explored to enhance the response and efficacy of RFA in combating PDAC. The figure emphasizes the importance of future directions in research to improve the outcomes and therapeutic options available for patients with PDAC *Note.* Adapted from “New Strategies for Treating Cancer”, by BioRender.com (2021). Retrieved from https://app.biorender.com/biorender-templates/figures/all/t-614cf572c1063f00a7681e74-new-strategies-for-treating-cancer

The complexity of the interaction between the microenvironment and cancer cells remains to be better characterized primarily in the context of emerging ablation therapies (Figure 4). The current standard of care for PDAC involving chemotherapy, radiation, and ICB has not yielded significant improvements in patient outcomes, underscoring a critical need for innovative treatment modalities. The integration of EUS-RFA into the therapeutic arsenal for PDAC presents a novel avenue for enhancing treatment efficacy, which implies that it could serve as an adjunct therapy to standard chemotherapy, potentially expanding the spectrum of effective treatment to a broader patient demographic. This approach may particularly benefit older or frail patients who are less likely to withstand aggressive treatments like high-dose chemoradiation. Table 2 lists current clinical trials incorporating EUS-RFA into the clinical management of pancreatic cancer.

Prospective clinical trials are crucial to validate the efficacy of EUS-RFA in combination with other therapeutic modalities for PDAC. These trials should focus on assessing the optimal timing, dosage, and sequence of EUS-RFA with ICB therapies and CD73 inhibitors. Moreover, understanding the long-term outcomes and potential adverse effects of these combination therapies is essential to ensure their safety and effectiveness.

In conclusion, EUS-RFA represents a transformative approach in the fight against PDAC. By harnessing the dual benefits of direct tumor ablation and systemic immune activation, EUS-RFA holds the potential to significantly improve patient outcomes.

## Abbreviations

ADA: adenosine deaminase
ADO: adenosine
AMP: adenosine monophosphate
AST: aspartate aminotransferase
C5/C5a: complement function
CD31: cluster of differentiation 31
CT: computed tomography
CTLs: cytotoxic T lymphocytes
CXCL12: chemokine (C-X-C motif) ligand 12
eADO: extracellular adenosine
EUS: endoscopic ultrasound
EUS-RFA: endoscopic ultrasound-guided radiofrequency ablation
FDA: Food and Drug Administration
FNA: fine needle biopsy
GZM: Granzyme B
HPLC: high-performance liquid chromatography
ICB: immune checkpoint blockade
IHC: immunohistochemical
IL: interleukin
IMC: imaging mass cytometry
INO: inosine
LAPC: locally advanced pancreatic adenocarcinoma
Ly6G: lymphocyte antigen 6 family member G
MDSCs: myeloid-derived suppressor cells
MPO: myeloperoxidase
mRNA: messenger ribonucleic acid
mTOR: mammalian target of rapamycin
OS: overall survival
PanCK+: pancytokeratin
PDAC: pancreatic ductal adenocarcinoma
PD-L1: programmed cell death ligand 1
TME: tumor microenvironment
Tregs: regulatory T cells
αSMA: α-smooth muscle actin

## Declarations

### Copyright

© The Author(s) 2024.
